# Oral Anticoagulant Treatment in Patients with Atrial Fibrillation and Chronic Kidney Disease

**DOI:** 10.3390/medicina57050422

**Published:** 2021-04-27

**Authors:** Mihai Ciprian Stoica, Zsolt Gáll, Mirela Liana Gliga, Carmen Denise Căldăraru, Orsolya Székely

**Affiliations:** 1 Department of Nephrology/Internal Medicine, Mures County Clinical Hospital, 540103 Târgu Mureș, Romania; mihai.stoica@umfst.ro (M.C.S.); mirela.gliga@umfst.ro (M.L.G.); carmen.caldararu@umfst.ro (C.D.C.); gall.orsolya525@gmail.com (O.S.); 2 Department of Pharmacology and Clinical Pharmacy, George Emil Palade University of Medicine, Pharmacy, Science and Technology of Târgu Mureș, 540142 Târgu Mureș, Romania

**Keywords:** oral anticoagulation, non-vitamin K oral anticoagulants, chronic kidney disease

## Abstract

Over the past few decades, a series of innovative medicines have been developed in order to optimize anticoagulation therapy for atrial fibrillation (AF). As a result, a number of nonvitamin K antagonist oral anticoagulants (NOAC) that directly target the enzymatic activity of factor II and factor Xa have been successfully licensed providing a more predictable effect and better safety profile compared to conventional anticoagulants (heparins and vitamin K antagonists (VKAs)). However, comparative efficacy and safety data is limited in patients with advanced chronic kidney disease (i.e., CKD stage 4/5 and end stage renal disease) because patients with estimated glomerular filtration rate (eGFR) < 30 mL/min/1.73 m^2^ were actively excluded from landmark trials, thus representing a major clinical limitation for the currently available agents. However, the renal function of AF patients can be altered over time. On the other hand, patients with CKD have an increased risk of developing AF. This review article will provide an overview of current concepts and recent evidence guiding the clinical use of NOACs in patients with CKD requiring chronic anticoagulation, and the associated risks and benefits of treatment in this specific patient population.

## 1. Introduction

Anticoagulation is the cornerstone of treatment in the prevention and management of thromboembolic diseases. With increasing prevalence reaching up to 15% among the elderly, atrial fibrillation (AF), is the most commonly encountered chronic arrhythmia, posing severe morbidity and mortality rates due to thromboembolic complications [1]. AF is associated with a series of cardiovascular comorbid conditions identified as clinical risk factors predisposing for stroke and systemic thromboembolism in trial cohorts. As a result clinical risk stratification scores have been widely implemented into practice, the CHA_2_DS_2_-VASc score being the most commonly utilized worldwide to detect patients with moderate or high risk of stroke and the need for oral anticoagulation [2].

**Figure 1:**
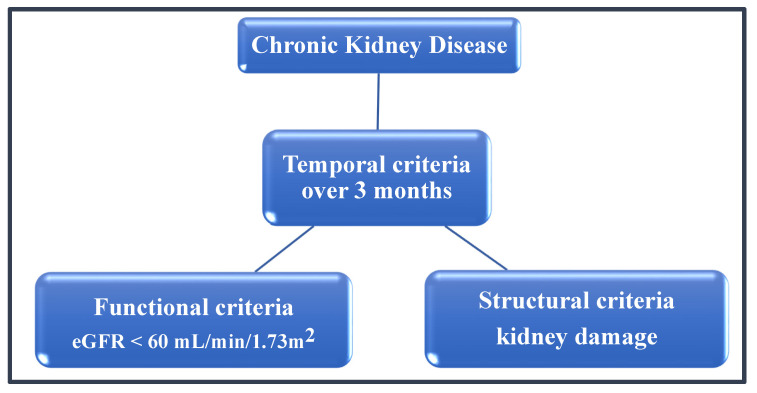
Diagnostic criteria for CKD.

Chronic kidney disease (CKD) is the term used for all stages of decreased kidney function, resulting from structural damage or functional nephron loss regardless of the underlying pathology that is persistent for more than three months [3] (for diagnostic criteria see Figure 1). The prevalence of CKD is increasing worldwide, reaching the maximum among the elderly with almost 700 million cases recorded internationally in 2017 and diabetic nephropathy accounting for around 30% of the underlying etiology [4]. The five main stages of CKD form a continuum as glomerular filtration rate (eGFR) progressively decreases (for the KDIGO classification see Table 1), with irreversible parenchymal and vascular glomerulosclerosis and consequent implications on health [5,6]; hence, guidelines are emphasizing the need for effective preventative measures, early detection, and treatment of CKD [3,7].

In the assessment of renal function estimations for eGFR derived from equations based on serum creatinine are routinely used in clinical practice. In everyday nephrology practice the most widely used equations for adult patients are the Chronic Kidney Disease Epidemiology Collaboration (CKD-EPI) and the Modification of Diet in Renal Disease (MDRD) Study equations. The CKD-EPI, developed in 2009, is considered the most reliable approximation of residual renal function and more accurate than the MDRD Study equation, particularly in people with higher levels of eGFR [8]. The Cockroft–Gault equation has been used for almost 50 years, in fact tendencies for overestimations have been reported in patients with advanced age and is no longer recommended for clinical use given the risk of overdosing drugs with narrow therapeutic range [9,10,11].

The accuracy of eGFR in the assessment of renal function is affected by non-steady-state conditions, serum creatinine levels altered by diet, muscle mass, etc.; therefore, other markers such as albuminuria are often considered [12]. However, there are limitations to the use of serum creatinine as no other biomarker has yet been able to supersede it [13].

## 2. Coexistence of AF and CKD

The prevalence of cardiovascular diseases and heart rhythm disorders, such as atrial fibrillation is significantly higher in patients with CKD than in the general population, which is important because these patients are more likely to die of an acute cardiovascular event (such as sudden cardiac death or myocardial infarction) rather than to develop end- stage renal disease (ESRD) [14,15]. The traditional cardiovascular risk factors, i.e., age, hypertension, obesity, diabetes mellitus, coronary artery disease, heart failure, and smoking are shared predisposing factors for AF and CKD. Both CKD and AF serve as trigger conditions to the other due to strong pathophysiologic interconnections and their coexistence is closely associated with poor long-term prognosis in affected patients [16,17].

Even in the early stages of CKD the cardiac remodeling processes are driven by the additive effect of (i) altered neurohormonal signaling (upregulated rennin–angiotensin–aldosterone system RAAS, and TGF-beta) and chronic sympathetic nervous system activation leading to hemodynamic overload [18]; (ii) proinflammatory mechanisms (increased circulating levels of CRP, TNF-α, fibrinogen, IL-6, and increased oxidative stress) [19]; and (iii) altered electrophysiology due to calcium handling abnormalities, decreased action potential, delayed conduction facilitating atrial re-entry pathways [20]. Consequently, the prevalence of AF is about two- to three-fold higher in CKD than in the general population [21]. Furthermore, the presence of AF is accelerating the progression of CKD to ESRD requiring renal replacement therapy secondary to its greater risk for heart failure, thromboembolic complications and cardiorenal syndrome [22].

On the other hand, there is an inverse relationship between eGFR and AF. There is strong evidence suggesting that the presence of micro- and macroalbuminuria is associated with increased risk of developing AF [23,24]. Similarly, a progressively worsening renal function is an independent thromboembolic risk factor in patients with AF. Thus, coexistence of both AF and CKD poses an elevated risk of stroke and all-cause mortality [25].

At the same time there is an elevated bleeding risk in CKD, explained by impaired platelet function secondary to uremic toxins, abnormal platelet arachidonic acid metabolism, altered function of the von Willebrand factor, intracellular adenosine and serotonin reduction, and the need for frequent invasive procedures [26,27].

Overall, CKD confers both thromboembolic and hemorrhagic risk at baseline. In addition, altered pharmacokinetics are leading to a massive challenge in the management of CKD patients in regard to oral anticoagulant treatment [28].

## 3. Oral Anticoagulation in Patients with Atrial Fibrillation—What Does Available Evidence Tell Us?

Polymorbidity and polypharmacy are contributing to an increasing burden in CKD and oral anticoagulants are among the top 15 drugs prescribed in patients with CKD for a variety of indications, such as thromboprophylaxis in AF and treatment or prevention of VTE [29].

VKAs, also called coumarins, have long served as the mainstay of long-term anticoagulation with proven efficacy, however, only providing clinical benefit if the anticoagulation effect is kept within the therapeutic range (INR 2.0–3.0). Their clinical use is challenging due to narrow therapeutic range, multiple drug–drug and food–drug interaction and the need for strict laboratory monitoring [30]. Although, recent evidence showed that INR self-testing can reduce some of the risks related to treatment [31], this may not apply to CKD patients.

As NOACs offer the relative efficacy, safety, and convenience compared with VKAs, they represent a major clinical advance and serve as suitable alternatives in oral anticoagulant therapy.

The meta-analysis including four pivotal phase III clinical trials demonstrated that NOACs are significantly associated with lower risk of stroke or systemic embolism (relative risks (RR) 0.81, 95% confidence interval (CI) 0.73–0.91; *p* < 0.0001), all-cause mortality (RR 0.90, 95% CI 0.85–0.95; *p* = 0.0003), and intracranial hemorrhage (RR 0.48, 95% CI 0.39–0.59; *p* < 0.0001) compared with warfarin [32]. Furthermore, Ando et al., conducted a subgroup analysis of the patients with AF and moderate CKD enrolled in the above-mentioned trials and concluded that NOACs showed lower incidence of both the ischemic endpoint and the major bleeding compared to warfarin [33]. An overview on phase III clinical trials demonstrating the safety and efficacy of NOACs for stroke prevention in patients with AF is provided in Table 2.

Despite the net clinical benefit over conventional anticoagulants, some pharmacodynamic and pharmacokinetic aspects of the NOACs still need to be considered. Undoubtedly, there is a lower potential for drug–drug and food–drug interactions compared to coumarins, but all the NOACs are substrate for the P-glycoprotein transporter, and rivaroxaban and apixaban are metabolized in the liver through the CYP-dependent isozyme pathway (CYP3A4) [38,39,40]. Thus, competitive inhibition of P-glycoprotein or CYP3A4 pathway will result in increased plasma levels of NOACs [41].

Although NOACs have a predictable pharmacokinetic profile with fixed daily doses without the need for anticoagulation monitoring, all of them are excreted by the kidney to some degree with the greatest renal dependency for dabigatran (see Table 3). Due to the dependence on renal clearance, the elimination of NOACs is reduced in patients with impaired renal function, potentially impacting efficacy and increasing bleeding risk [29,42,43]. Although the phase III trials excluded patients with severe renal impairment of a creatinine clearance (CrCl) < 25 to 30 mL/min, some cohort studies have demonstrated that NOACs also provide effective thromboprophylaxis in AF patients with mild to moderate renal dysfunction (CrCl of 30–79 mL/min) [44].

A subgroup analysis from the ARISTOTLE trial evaluated the safety of apixaban compared to warfarin in 269 patients with AF and advanced CKD (CrCl 25–30 mL/min). Stanifer et al. found that apixaban caused less bleeding than warfarin, with even greater reductions in bleeding than in patients with CrCl > 30 mL/min [46].

## 4. The Use of NOACs in Patients with AF and Concomitant CKD

There is increasing evidence on the use of NOACs in individuals with mild-to-moderate CKD; however, their safety and efficacy are yet to be proved in advanced stages of CKD. The most commonly used study model, certainly the most relevant to clinical practice is AF in patients with CKD.

A 2019 meta-analysis that included 45 randomized trials on oral anticoagulation strategies, total number of 34,000 patients, most of them with AF and associated mild-to-moderately impaired kidney function, reported a statistically significant benefit over warfarin in reducing the risk of stroke (risk ratio (RR), 0.79; 95% CI 0.66–0.93), without an obvious increase in bleeding (RR for major bleeding, 0.80; 95% CI 0.61–1.04; RR for intracranial hemorrhage (ICH), 0.49; 95% CI 0.30–0.80) and a trend towards improved survival (RR, 0.88; 95% CI 0.78–0.99). Individuals with end-stage renal disease (eGFR < 15 mL/minute/1.73 m^2^ or creatinine clearance (CrCl) < 20 mL/min) were mostly excluded, and the evidence is scant to recommend either class of oral anticoagulation for better outcomes [47].

A retrospective cohort study conducted in 2018 through United States Renal Data System included around 25,000 patients with ESRD and AF undergoing hemodialysis who were initiated on oral anticoagulant treatment. The study population was matched to apixaban versus warfarin cohort in 1:3 ratio, other NOACs were underrepresented. According to Siontis and colleagues there was no difference in the risk of stroke and systemic thromboembolism between apixaban and warfarin (HR, 0.88; 95% CI, 0.69–1.12; *p* = 0.29), but apixaban was associated with a significantly lower risk of major bleeding (HR, 0.72; 95% CI, 0.59–0.87; *p* < 0.001). Furthermore standard dose of Apixaban 5 mg BD was associated with significantly lower risks of stroke/systemic embolism and death as compared with either reduced-dose apixaban (2.5 mg twice a day; *n* = 1317; HR, 0.61; 95% CI, 0.37—0.98; *p* = 0.04 for stroke/systemic embolism; HR, 0.64; 95% CI, 0.45–0.92; *p* = 0.01 for death) or warfarin (HR, 0.64; 95% CI, 0.42–0.97; *p* = 0.04 for stroke/systemic embolism; HR, 0.63; 95% CI, 0.46–0.85; *p* = 0.003 for death) [48].

A 2020 systematic review that included nine studies (two of which were randomized trials) of individuals with AF or VTE who had CKD or were receiving dialysis found similar efficacy with NOACs versus warfarin and similar bleeding risks with apixaban versus warfarin [49].

Recently published data from the Polish Atrial Fibrillation (POL-AF) Registry, a nationwide prospective observational study on NOAC prescribing trends in patients with AF, found that 50.3% of the study population had some degree of renal disease (defined as eGFR < 60 mL/min) and Apixaban was the preferred drug of choice in this patient subgroup [50].

## 5. Current International Guidelines for Oral Anticoagulation Treatment in CKD

There is a huge debate and lack of consensus between the international guidelines’ recommendation with regards to the use of oral anticoagulants in patients with AF and advanced CKD.

The international guideline group Kidney Disease Improving Global Outcomes KDIGO 2012 guideline indicates lower doses of warfarin with close monitoring when eGFR < 30 mL/min. Routine anticoagulation in patients with CKD stage 5 on dialysis is not recommended for primary prevention of stroke. However, the aforementioned guidelines date from 2012, and therefore these recommendations should be followed with caution [15].

According to the European Society of Cardiology (ESC) 2020 Guidelines for Management of Atrial Fibrillation anticoagulation can be safely used in AF patients with concomitant moderate and moderate-to-severe CKD, i.e., eGFR > 15 mL/min/1.75 m^2^. The use of VKA is proven to be beneficial in reducing the risk of systemic thromboembolism, however, poses a significantly increased risk of bleeding. Thus, assessment of the individual patient risks and regular monitoring of renal function is crucial to guide dose adjustments of NOACs [51]. It is important to note, that none of the NOACs have been approved in Europe for patients with CrCl < 15 mL/min or on dialysis [45].

The American College of Cardiology/American Heart Association/Heart Rhythm Society (ACC/AHA/HRS) guidelines from 2019 state that warfarin and apixaban may be used without dose restrictions when CrCl < 15 mL/min and regardless of the need for RRT. However other NOACs to be avoided in ESRD patients and on RRT (i.e., when CrCl falls below 15 mL/min) due to lack of evidence for benefits [52].

In contrast the Canadian Cardiovascular Society (CCS) updated guidelines from 2020 suggest that warfarin is recommended with eGFR 15–30 mL/min and not on dialysis, but patients with AF receiving dialysis should not be prescribed oral anticoagulation or aspirin for stroke prevention [53,54].

The CHEST 2018 guideline and expert panel report from the American College of Chest Physicians recommends VKAs and selected reduced dose NOACs (rivaroxaban 15 mg QD, apixaban 2.5 mg bid, edoxaban 30 mg QD and (in USA only) dabigatran 75 mg bid) to be used with caution in CKD stage IV (CrCl 15–30 mL/min). In ESRD (CrCl < 15 mL/min or dialysis-dependent), NOACs should generally not be used, but well-managed VKA with time-in-therapeutic range (TTR) > 65 to 70% (ungraded consensus-based statement) and individualized decision-making applies [55].

Even though, in 2018 the United States Food and Drug Administration has extended apixaban use to patients with advanced CKD and hemodialysis, apixaban use for stroke prophylaxis among patients with AF and ESRD is becoming popular. However, the evidence behind this approach is limited to only pharmacokinetic studies [56,57]. A recent network meta-analysis raised concerns regarding effectiveness with the dosage of 2.5 mg twice daily [58].

**Figure 2:**
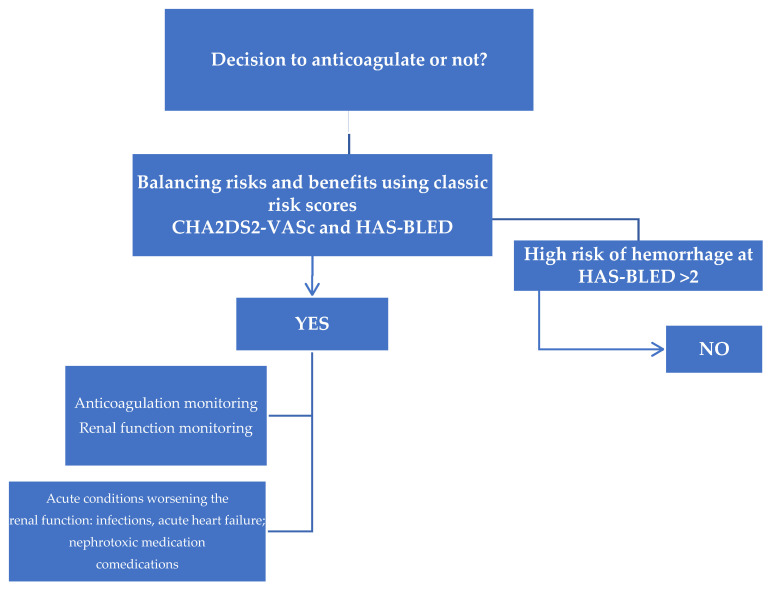
Individualized approach for decision to anticoagulate or not in patients with AF and advanced CKD.

Altogether, the currently available recommendations are controversial and definitive clinical guidelines derived from randomized controlled trials are urgently needed to aid clinical decision making in this complex and highly comorbid patient population. Until then clinicians are left with the need of a personalized approach weighing the risks and benefits associated with anticoagulation (Figure 2).

## 6. Currently Ongoing Studies on Oral Anticoagulation in ESRD

In order to establish safe practice of anticoagulation in patients with ESRD and concomitant AF there are some recently completed and ongoing trials, comparing the efficacy and safety of apixaban versus warfarin, as well as randomized studies focusing on the hemorrhagic and thrombotic risks associated with oral anticoagulation treatment (OAT) versus no anticoagulation (see Table 4). The recently completed Renal Hemodialysis Patients Allocated Apixaban Versus Warfarin in Atrial Fibrillation trial (RENAL-AF; ClinicalTrials.gov identifier NCT02942407) was an open-label randomized study, with the goal of assessing the safety and efficacy of apixaban for stroke prophylaxis among patients with AF and ESRD on hemodialysis. However, it had to be stopped early due to limited resources. The preliminary results of this trial indicate that apixaban 5 mg BID results in similar rates of bleeding and strokes as warfarin among patients with ESRD on hemodialysis. An important limitation of this study is the suboptimal TTR within the warfarin group (approximately 44%) with a large proportion of patients in the subtherapeutic range. It remains unclear if lower apixaban dose (2.5 mg BID) and cessation of aspirin (used in ~40%) would have resulted in lower bleeding rates compared with warfarin (Pokorney SD. Renal hemodialysis patients allocated apixaban versus warfarin in atrial fibrillation—RENAL-AF- presented at the American Heart Association Annual Scientific Sessions; 16 November 2019; Philadelphia, PA, USA).

All other currently registered prospective trials are in recruitment phase. The Compare Apixaban and Vitamin-K Antagonists in Patients with Atrial Fibrillation and End-Stage Kidney Disease (AXADIA; ClinicalTrials.gov identifier NCT02933697) trial is comparing phenprocoumon and apixaban (with a planned enrollment of 222 patients); Oral Anticoagulation in Hemodialysis Patients (AVKDIAL ClinicalTrials.gov identifier NCT02886962) trial is assessing bleeding risks in regards to VKA versus no OAT with a target of 855 patients and the Strategies for the Management of Atrial Fibrillation in Patients Receiving Dialysis (SAFE-D; ClinicalTrials.gov identifier NCT03987711) trial, comparing warfarin, apixaban, and no anticoagulation (with a planned enrollment of 150 patients).

A prospective multicenter noninterventional real-world European registry is currently ongoing (Factor XA-Inhibition in REnal Patients with Non-valvular Atrial Fibrillation-XARENO) collecting clinical data from 2500 patients with AF and CKD (eGFR 15–49 mL/min) receiving anticoagulation at the discretion of the clinician based on the currently available guidelines (Rivaroxaban, VKA or no OAC) with the aim to assess CKD progression and clinical outcomes in regards to anticoagulation strategies in everyday clinical practice (ClinicalTrials.gov identifier NCT02663076).

## 7. Conclusions

To summarize the currently available evidence-based recommendations in patients with concomitant thromboembolic risk or disease and advanced CKD we could formulate a general approach as follows:

- In mild-to-moderate CKD (eGFR 30–50 mL/min or higher), the registry evidence discussed above suggests that NOACs are preferred options over VKAs for both efficacy and safety. Dose adjustments may be appropriate as directed for the specific agents.
- In severely impaired kidney function (eGFR < 30 mL/min), there is limited evidence to predict how NOACs may compare VKAs, although evidence for superior efficacy and safety over warfarin continues to accumulate. There is no RCT based evidence to support anticoagulation therapy in ESRD. OAT should only be initiated after careful consideration of benefit and harm. Warfarin is generally preferred over a NOAC in patients who require long-term anticoagulation.
- In AF patients that had a history of major bleeding and contraindications to OAT, catheter-based occlusion of the left atrial appendage could be considered.

The advanced CKD and ESRD population represent a unique challenge in the clinical practice when anticoagulation is indicated either for stroke and systemic embolism prevention in AF or in the treatment of VTE. This highly complex and comorbid population is at increased risk of both thromboembolism and major bleeding at baseline, further complicated by the altered pharmacokinetics and renal dependency of the currently available NOACs.

The currently available international guidelines are contradictory. In order to improve clinical outcomes and achieve the best management approach further prospective studies are urgently needed.

Whilst awaiting the results from randomized clinical trials, the decision to anticoagulate or not in advanced kidney disease remains a challenging task for the clinician. Perhaps an individualized holistic approach taking into account the patient’s clinical risk factors, compliance, treatment adherence and preferences is advisable but remembering the principle of “primum non nocere” must remain in focus. It should be pointed out that dialyzed patients are every-other-day anticoagulated during the dialysis sessions. The blood pressure variations and other factors influencing the hemorrhagic risk can be restrictive indications for NOACs or even VKAs.

## Figures and Tables

**Table 1:** The stages of chronic kidney disease ^1^.

CKD	eGFR (mL/min/1.73 m^2^)	A1 < 30 mg/g	A2 30–300 mg/g	A3 > 300 mg/g
Stage 1	>90	A0	A1	A2
Stage 2	60–89	A0	A1	A2
Stage 3a	45–59	A1	A2	A3
Stage 3b	30–44	A2	A3	A3
Stage 4	15–29	A3	A3	A3
Stage 5	<15	A3	A3	A3

^1^ Adopted from KDIGO [3]. eGFR—estimated glomerular filtration rate; A1–3—degree of albuminuria.

**Table 2:** Phase III clinical trials demonstrating the safety and efficacy of NOACs compared to VKA for stroke prevention in patients with AF.

Clinical Trial	Author, Year	Study Population	Study Design	Results Primary Outcome: Stroke or Systemic Embolism (SSE) Safety Outcome: Major Bleeding or Clinically Relevant Major Bleeding
RE-LY	Connolly S. et al., 2009 [34]	*n* = 18,113; CHADS2 ≥ 1; 71 years, 64% men	Dabigatran 110 mg/150 mg twice a day compared to dose-adjusted warfarin 2 years follow-up	Dabigatran 110 mg was noninferior to warfarin with lower rate of ICH and other major hemorrhage; Dabigatran 150 mg was superior to warfarin with lower rate of ICH, similar rate of other major hemorrhage
ROCKET AF	Patel et al., 2011 [35]	*n* = 14,264; CHADS2 ≥ 2; 73 years, 60% men	Rivaroxaban 20 mg (15 mg in patients with moderate renal impairment) once a day compared to dose-adjusted warfarin	Rivaroxaban was noninferior to warfarin with lower rate of ICH, similar rate of other major hemorrhage The reduced dosage showed consistent results with 20 mg once daily in patients with normal renal function
ARISTOTLE	Granger et al., 2011 [36]	*n* = 18201; CHADS2 ≥ 1; 70 years, 65% men	Apixaban 5 mg (2.5 mg in patients with two or more dose-reduction criteria) twice a day compared to dose- adjusted warfarin	Apixaban was superior to warfarin with lower rate of ICH and lower rate of other major hemorrhage The treatment effect and major bleeding were consistent across all major subgroups
ENGAGE AF-TIMI 48	Gugliano et al., 2013 [37]	*n* = 21,105; CHADS2 ≥ 2; 72 years, 62% men	Edoxaban 30 and 60 mg once a day compared to dose- adjusted warfarin	Both once-daily regimens of edoxaban were noninferior to warfarin with respect to the prevention of SSE and with significantly lower rates of bleeding side effects

ICH—intracranial hemorrhage; SSE—stroke or systemic embolism; Of note: Patients with advanced CKD (CrCl < 25 to 30 mL/min) were actively excluded.

**Table 3:** Pharmacodynamic and pharmacokinetic properties of commonly used oral anticoagulants.

Characteristics	Warfarin	Dabigatran	Rivaroxaban	Apixaban	Edoxaban
Mechanism of action	Inhibition of vitamin K dependent clotting factors (II, VII, IX, X)	Factor IIa (thrombin) inhibition	Factor Xa inhibition	Factor Xa inhibition	Factor Xa inhibition
Dosing	Variable (INR monitoring) QD	Fixed 150/110 mg BID	Fixed 20/15 mg QD	Fixed 5/2.5 mg BID	Fixed 60/30 mg QD
Protein binding	99%	35%	90%	87%	40–59%
Metabolism	Extensive metabolism by CYP2C9	Esterase mediated hydrolysis (no CYP450)	Metabolized in liver by CYP3A4/ 2J2 (65%)	Metabolized in liver by CYP3A4 (75%)	Metabolized in liver by CYP3A4 (50%)
Interactions	Multiple food-drug and drug-drug	P-gP	CYP3A4/2J2 P-gP	CYP3A4 P-gP	P-gP
Renal excretion	<1%	80–85%	35%	25%	50%
C_max,_ hours	72–96	1–2	2–4	3–4	1–2
t_½_, hours	40	12–14	6–13	12	10–14
Dialyzable	No	Yes	No	Small	No
Antidote	Yes (Vitamin K)	Yes (Idarucizumab)	Yes (Andexanet alfa)	Yes (Andexanet alfa)	Under development
Recommendation in severe renal impairment (eGFR = 15–29 mL/min/1.73 m^2^)	Strict INR monitoring	Contraindicated (EU)/Dose adjustment (75 mg BID (US))	Dose adjustment (15 mg QD)	Dose adjustment (15 mg QD in EU)/No action until at least 2 criteria fulfilled (age ≥ 80 y; weight ≤ 60 kg; creatinine ≥ 1.5 mg/dL)	Dose adjustment (30 mg QD)

Adopted from [29,38,39,40,45]. Legend: QD—once a day; BID—twice a day; P-gP—P glycoprotein transporter involved in absorption and renal clearance—plasma levels may be influenced by P-gP inducers or inhibitors; CYP450—cytochrome P 450 CYP3A4 involved in hepatic clearance—plasma levels may be affected by CYP3A4 inducers or inhibitors; Cmax—peak concentration; t1/2—half-life; INR—international normalized ratio.

**Table 4:** Clinical trials evaluating the efficacy and safety of oral anticoagulation in patients with end-stage renal disease and concomitant atrial fibrillation.

Clinical Trial	Study Design/Enrollment	Methods	Inclusion Criteria	Primary and Secondary End-Point	Expected Completion/Results
RENAL-AF (NCT02942407)	2016 Apixaban pharmacokinetics US, Multicenter (*n* = 762 patient target)	Open-label randomization to apixaban (5/2.5 mg) versus warfarin (INR 2–3) for up to 15 months	≥18 years AF with CHA2DS2-VASc ≥ 2 ESRD on HD > 3 months OAT candidate	Time to first major or clinically relevant non-major bleeding Stroke or SE Mortality	August 2019 (154 patients enrolled at completion); Similar risks of bleeding and stroke in the 2 groups
AXADIA (NCT02933697)	2017 Apixaban pharmacokinetics Germany, Multicenter (*n* = 222 patient target)	Open-label randomization to apixaban (2.5 mg) versus phenprocoumon (INR 2–3) for 6–24 months	≥18 years AF with CHA2DS2-VASc ≥ 2 ESRD on HD > 3 months OAT candidate	Time to first major or clinically relevant non-major bleeding Thromboembolism	July 2022 (Recruiting)
AVKDIAL (NCT02886962)	2017 University Hospital of Strasbourg, France, Multicenter (*n* = 855 patient target)	Open-label randomization to VKA (INR 2–3) versus no OAT for 24 months	≥18 years AF with CHA2DS2-VASc ≥ 2 ESRD on HD > 1 months OAT candidate	Cumulative incidence of severe bleeding events and thrombosis	January 2023 Recruiting
SAFE-D (NCT03987711)	2019 Unity Health Toronto, Multicenter (*n* = 150 patient target)	Open-label randomization to apixaban (5/2.5 mg) versus warfarin (INR 2–3) versus no OAT for 26 weeks	≥18 years AF with CHA2DS2-VASc ≥ 2 ESRD on HD > 90 days OAT candidate	AF related stroke and SE; risk of bleeding and all-cause mortality	December 2021 Recruiting

CKD—chronic kidney disease; ESRD—end-stage renal disease; AF—atrial fibrillation; OAT—oral anticoagulation treatment; VKA—vitamin K antagonist; INR—international normalised ratio; SE—systemic embolism; HD—hemodialysis.
